# Modeling the kinetics of heteromeric potassium channels

**DOI:** 10.3389/fncel.2022.1036813

**Published:** 2022-11-10

**Authors:** Kees McGahan, James Keener

**Affiliations:** Math Department, University of Utah, Salt Lake City, UT, United States

**Keywords:** heteromeric potassium ion channels, *K_v_*1 channels, *K_v_*7 channels, mathematical modeling, ion channel kinetics

## Abstract

Mechanistic mathematical modeling has long been used as a tool for answering questions in cellular physiology. To mathematically describe cellular processes such as cell excitability, volume regulation, neurotransmitter release, and hormone secretion requires accurate descriptions of ion channel kinetics. One class of ion channels currently lacking a physiological model framework is the class of channels built with multiple different potassium protein subunits called heteromeric voltage gated potassium channels. Here we present a novel mathematical model for heteromeric potassium channels that captures both the number and type of protein subunits present in each channel. Key model assumptions are validated by showing our model is the reduction of a Markov model and through observations about voltage clamp data. We then show our model's success in replicating kinetic properties of concatemeric channels with different numbers of *K*_*v*_1.1 and *K*_*v*_1.2 subunits. Finally, through comparisons with multiple expression experiments across multiple voltage gated potassium families, we use the model to make predictions about the importance and effect of genetic mutations in heteromeric channel formation.

## 1. Introduction

Potassium channels are membrane spanning proteins that are responsible for the transport of potassium ions into and out of cells. Found in virtually all species, these channels regulate a whole host of cellular processes (MacKinnon, 2003). In addition to their importance in regulating neuron, cardiac cell, and pancreatic β-cell excitability, potassium channels have been found to aid in other physiological functions such as volume regulation, neurotransmitter release, and hormone secretion (Barfield et al., 2005; Ghatta et al., 2006). Working to account for all these function are upwards of 80 genes coding for different potassium channel protein α-subunits (Harding et al., 2022). These are categorized into three broad groups based on their number of transmembrane domains (TMDs): inward rectifier channels (two TMDs), leak or *K*_2*p*_ channels (four TMDs), and voltage gated or *K*_*V*_ channels (six TMDs) (Coetzee et al., 1999).

The most well-studied of these groups, accounting for about half of the total genes, is the collection of voltage gated potassium channel subunits. Broken up into 12 subfamilies according to physiological characteristics, four *K*_*V*_ α-subunits are required to form a functional, pore forming channel. Each of the four α-subunits can be encoded for by one gene forming a homotetramer (homomer), or by multiple genes from either the same, or in rare cases, different subfamilies thereby forming heterotetramers (heteromers). As a result of all the different possible numbers and arrangements of each subunit type, formation of these heteromeric potassium channels drastically increases *K*_*V*_ channel diversity. With such a large collection of possible channels, researchers have begun asking which subunit combinations are physiologically relevant and how do their kinetics compare to their homomeric counterparts?

Attempts at answering these questions have taken a variety of forms including using pharmacological and electrophysiological techniques, and studying properties of concatenated subunit genes and expression systems. Work by Cordeiro et al. (2019) concatenated different ratios of *K*_*V*_1 subunits together and examined the concatemers' different levels of binding affinity to κM-conotoxin RIIIJ. It was found that κM-conotoxin RIIIJ not only had preference for channels with *K*_*V*_1.1, *K*_*V*_1.2, and *K*_*V*_1.6 subunits, but had the highest binding affinity for heteromers in the particular configuration of three *K*_*V*_1.2 subunits and 1 *K*_*V*_1.1 or *K*_*V*_1.6 subunit. Al-Sabi et al. (2013) combined electrophysiology tools with *K*_*V*_1.1/*K*_*V*_1.2 concatemers and determined distinct activation kinetics and TEA sensitivity distinguishing each of the heteromeric and homomeric concatemers. While looking at epilepsy associated mutations in *K*_*V*_7.2 genes, Miceli et al. (2013, 2015) showed that wildtype and mutant *K*_*V*_7.2 subunits likely form heteromeric channels whose kinetic properties are intermediate between those of the associated homomeric channels. Similar conclusions were reached about the heteromers formed from *K*_*V*_1.1, *K*_*V*_1.2, and *K*_*V*_1.1 mutant subunits thought to be responsible for ataxia and epilepsy (Hasan et al., 2017; Miceli et al., 2022).

One of the current problems faced in this field of work is connecting the results of these concatenation experiments with experiments involving natural expression systems. Determining which heteromeric ratio of subunits is present in a natural expressed system has remained elusive for experimentalists. The straightforward technique of gene knockouts renders all functioning, present heteromers useless thereby preventing further analysis. One possible avenue of approach to this issue is mathematical modeling. Since the groundbreaking work of Hodgkin and Huxley on modeling action potentials in the squid giant axon, mechanistic math modeling has become a staple in furthering understanding of homomeric ion channels and their role in cellular processes (Hodgkin and Huxley, 1952; Miura, 2002; Moreno et al., 2016; McGahan and Keener, 2020). Two types of general model frameworks are typically considered to describe homomeric ion channel function: those in the spirit of Hodgkin and Huxley, and Markov models that explicitly detail each conformational protein change between channel states (Nekouzadeh et al., 2008; Keener, 2009, 2010). Increases in data availability and advancements in experimental techniques have led to more detailed models capable of answering and asking questions about drug-channel interactions, complex experimental protocols, different activating stimuli, and even channel structure (Cheng et al., 2007; Perissinotti et al., 2015; Moreno et al., 2016).

Successful modeling results, across multiple ion channel types, hints at modeling as a natural next step for connecting the different types of existing heteromeric *K*_*V*_ channel experimental results. Models of heteromeric channels, especially biophysical ones reflecting protein composition and structure, are currently scarce relative to the available data and experimental interest. Work by Cheng et al. (2007) coupled spectroscopy-based fluorescence resonance energy transfer and computational modeling to indicate that heat-sensitive transient receptor potential channels prefer heteromeric configurations. Looking at heteromeric cyclic nucleotide–gated channels, Benndorf et al. (2022) analyzed the ability to determine model parameters using concatemeric experimental data. Modeling work with heteromeric *K*_*V*_ channels has primarily consisted of fitting Boltzmann-like equations, or in rare cases Markov Models, for channel gating to voltage clamp data of either concatemeric heteromers or cells with two or more coexpressed cDNA types (Sale et al., 2008; Miceli et al., 2013, 2015). Although these fitted heteromeric *K*_*V*_ models can be used to simulate generic heteromeric behavior, they do not encode knowledge about the α-subunit ratios. This limitation implies they cannot be generalized to heteromers of different ratios let alone heteromeric channels with completely different subunits.

Using a Hodgkin-Huxley type gating model, which we show has a parallel Markov structure, we present a novel heteromeric *K*_*V*_ channel modeling framework. This framework is both generalizable to multiple *K*_*V*_ families and captures α-subunit type and stoichiometry. We outline the applicability of our model and justify specific model assumptions. With *K*_*V*_1.1/*K*_*V*_1.2 concatemeric results from Al-Sabi et al. (2013) we analyze the model's ability to replicate heteromeric channels' steady state open probability curves for each of the possible subunit combinations. We then apply our framework to look at multiple different experiments where two cDNAs for *K*_*V*_ subunits are expressed together but the subunit ratios and relative percentages of the resulting channels is unknown. By comparing these known experimental results with model outputs we make predictions about which subunit ratios are important and when α-subunits may be assembling randomly.

## 2. Methods

### 2.1. Hodgkin Huxley model framework

Our model is based on the work of Hodgkin and Huxley (1952). Their model tracks the membrane voltage *V*, which changes based on the sum of the individual ionic currents. The model for each current is composed of three parts: a maximal conductance *g*_*i*_, an open probability consisting of some number of activating or inactivating gates, and a driving force term (*V*−*E*_*i*_) describing the direction and magnitude of the current, for *i* = *Na, K*. The gating variables, *n, m*, and *h*, are each probabilities between 0 and 1, that change over time in response to changes in *V*. It is assumed that the gates operate independently and that all gates must be open for current flow. The numbers of each gate for each channel type were derived by Hodgkin and Huxley based on best fits to data. The differential equations for membrane voltage *V*, the sodium gates *m, h*, and the potassium gates *k* are given here:


(1) $$\begin{array}{l} \frac{dV}{dt}=\frac{1}{Cm}\left(-I_{Na}-I_{K}-I_{l}\right) \end{array}$$

(2) $$\begin{array}{l} \frac{dj}{dt}=\left[a_{j}\left(V\right)\left(1-j\right)-b_{j}\left(V\right)j\right],j=m,n,h. \end{array}$$

With the Hodgkin-Huxley current equations having the form:


(3) $$\begin{array}{l} I_{Na}=g_{Na}m^{3}h\left(V-E_{Na}\right) \end{array}$$

(4) $$\begin{array}{l} I_{K}=g_{K}n^{4}\left(V-E_{K}\right) \end{array}$$

(5) $$\begin{array}{l} I_{l}=g_{l}\left(V-E_{l}\right). \end{array}$$

Rate constants *a*_*j*_, *b*_*j*_, and parameters, including the maximal conductances *g*_*K*/*Na*/*L*_, reversal potentials *E*_*K*/*Na*/*L*_, and membrane capacitance *Cm* were all experimentally fit and are available in the literature (Hodgkin and Huxley, 1952; Keener and Sneyd, 2009; McGahan and Keener, 2020).

### 2.2. Heteromeric potassium channels (activating only)

Here we formalize our model structure proposed for heteromeric *K*_*V*_ channels. This paper focuses on the set of heteromeric channels whose α-subunits, when in homomeric configurations, confer minimal, or irrelevant inactivating kinetics. Each homomeric channel is modeled identically to the Hodgkin Huxley potassium channel, with the necessary rate constants. Each heteromeric channel is also mathematically described with three components as outlined above in Section 2. While the density and driving force are modeled identically to a homomeric potassium channel (Equation 4), the channel gating of a heteromer is modeled to reflect the specific number and type of α-subunits present.

Since each *K*_*V*_ channel, homomeric and heteromeric, is composed of four protein subunits, we assume that each heteromer has 4 gates, with number and type equal to that of the subunits present (Coetzee et al., 1999; Cordeiro et al., 2019). The gating variables and corresponding differential equations used in any heteromeric model are taken directly from their respective homomeric models. This guarantees that a heteromer's properties are derived mechanistically and are completely determined from subunit composition once the homomeric kinetics are known. As an example, to model a 1.1/1.2 K-channel heteromer, notated by either *K*_*V*_1.1/1.2 or *K*_1.1/1.2_, we first need *K*_*V*_1.1 and *K*_*V*_1.2 homomer models that are built with four independent and identical gates, *n*_1.1_ and *n*_1.2_. These homomeric models are referred to as the 4:0 and 0:4 models, denoting their subunit, and thus gate, number, and type. To then construct a *K*_*V*_1.1/1.2 heteromer consisting of *x* 1.1 subunits and *y* 1.2 subunits, the model is referred to as an *x*:*y* model and is built using *x* 1.1 gates and *y* 1.2 gates.

The representation for a heteromeric channel current made up of three 1.1 subunits and one 1.2 subunit is given below:


(6) $$\begin{array}{l} I_{1.1}=g_{1.1}n_{1.1}^{4}\left(V-E_{K}\right) \end{array}$$

(7) $$\begin{array}{l} I_{1.2}=g_{1.2}n_{1.2}^{4}\left(V-E_{K}\right) \end{array}$$

(8) $$\begin{array}{l} I_{1.1/1.2}^{3:1}=g_{1.1/1.2}n_{1.1}^{3}n_{1.2}^{1}\left(V-E_{K}\right). \end{array}$$

As stated above, it is assumed *x* + *y* = 4 with *x* and *y* taking integer values between 0 and 4. This is to adhere to the 1 subunit for 1 gate hypothesis. We emphasize that setting either *x* or *y* to 0 returns a homomeric channel. Note that loosening the integer value restriction does not break the resulting analysis below, it simply carries a different set of biological assumptions with it.

### 2.3. Addressing the four gate and integer power assumptions

The primary model assumptions to address are that the sum of the number of gates must be 4 and that the gates take integer power values. These assumptions inherently dictate that each homomeric K-Channel is modeled with four gates. While Hodgkin and Huxley's original model utilized four gates to fit the data, since then there have been many activating only, homomeric, potassium channel models in the literature fit with only one activating gate (Hodgkin and Huxley, 1952; Miceli et al., 2013, 2015; Ranjan et al., 2019). There are two arguments outlined here to defend these two critical model assumptions. The first is that this model framework is actually a reduction of a well described Markov model for four interacting independent protein subunits of two (or more) types. The second argument involves looking at the outputs of voltage clamp recordings of cells containing *K*_*v*_1.1 channels and seeing which number of gates best fits the time dependent data.

#### 2.3.1. Markov model reduction

Work by Keener takes Markov models of ion channels and finds globally attracting invariant manifolds of reduced dimensions (Keener, 2009, 2010). The first important result of these papers is for a potassium channel with four identical, independent subunits that can only transition between an open or closed state. If the conducting state is when all four subunits are open, then the Markov model's invariant manifold has exactly Hodgkin and Huxley's open probability of $P_{o}=n^{4}$, with *n* governed by Equation (2). This Markov model and the invariant manifold's open probability are precisely what we have postulated for all homomeric potassium channel models under our outlined framework.

The second important result by Keener is for a sodium channel (Keener, 2009, 2010). The given Markov model has three activating subunits and 1 inactivating subunit, with all subunits independent. The model is shown to have an invariant manifold with open probability $P_{o}=m^{3}h$ for *m* and *h* governed by Equation (2). As seen with Equation (8), the open probability for the reduced sodium model $P_{o}=m^{3}h$, is equivalent to the open probability for a 3:1 or 1:3 heteromer. In both cases, there are three subunits of 1 type, 1 subunit of a different type, and all subunits must open to be conducting. Therefore, this result by Keener implies our 3:1 and 1:3 heteromeric models can be thought of as an invariant manifold of a Markov model for 3 subunits of one K-channel subunit type and 1 subunit of a second type, all independent. Finally, although not explicitly detailed, the manuscript posits that a Markov model with two independent subunits of type *K*_*v*_1.1 and *K*_*v*_1.2, would have an invariant manifold with open probability $P_{o}=n_{1.1}^{2}n_{1.2}^{2}$, again what our framework predicts. One important observation is that this is the predicted open probability for a 2:2 heteromer regardless of if subunits of the same type are across from or next to each other.

#### 2.3.2. Fitting to voltage clamp data

The second justification for our model assumptions is done by looking at voltage clamp data for *K*_*v*_1.1 channels. Assuming that our homomeric potassium currents can be accurately modeled with a maximal conductance *g*_*k*_, an open probability determined by some unknown number *p* of gates of type *n*, and a driving force term (*V* − *E*_*k*_); we have the current equation:


(9) $$\begin{array}{l} I_{K}=g_{K}{n\left(t\right)}^{p}\left(V-E_{K}\right). \end{array}$$

Using voltage clamp data for cells with *K*_*v*_1.1 channels we ask which power *p* best describes the channel gating. The first step in answering this question is to normalize any endogenous or capacitive currents that may be present in the data. This is done by subtracting the current value that is present at the very start of the voltage clamp protocol since before the channels have had time to activate there should be no noticeable current. This is all to say that the current traces at the initial engagement of the protocol should start with a current reading of 0 with some given noise. Next, since voltage is fixed, we can divide out the driving force term (*V* − *E*_*K*_) if *E*_*K*_ is known. Then, assuming the voltage clamp has been performed at a high enough voltage to open all gates giving *n*(*t*)^*p*^ = 1, we can divide by the maximum value attained over the entire experiment. This step corresponds to dividing out *g*_*K*_. The resulting transformed data is a function of time whose output is the open probability of the present *K*_*v*_ channels. This data will be fit with the function for the open probability *P*_*o*_(*t*):


(10) $$\begin{array}{l} P_{o}\left(t\right)=n{\left(t\right)}^{p}. \end{array}$$

which is what is left from Equation (9) after dividing by the maximal conductance *g*_*K*_ and driving force term (*V* − *E*_*K*_).

To fit Equation (10) to the transformed data we also must know the form *n*(*t*) takes at any fixed voltage. Recall that the differential equation governing a single gate's dynamics is described as follows:


(11) $$\begin{array}{l} \frac{dn}{dt}=\left[a_{n}\left(V\right)\left(1-j\right)-b_{n}\left(V\right)n\right]=\frac{n_{\infty }-n\left(V\right)}{\tau_{n}} \end{array}$$

where $n_{\infty }\left(V\right)=\frac{a_{n}}{a_{n}+b_{n}}$ and $\tau_{n}\left(V\right)=\frac{1}{a_{n}+b_{n}}$. Under this formulation *n*_∞_ describes the steady state open probability attained by the gate as a function of voltage, and τ_*n*_ is the voltage dependent time constant. If the channel behavior is analyzed under a voltage clamp protocol with voltage kept constant, then the resulting ODE is dependent only on *n*. Therefore, Equation (11) can be solved analytically to yield the following expression in *n*:


(12) $$\begin{array}{l} n\left(t\right)=n_{\infty }\left(1-Ae^{\frac{-t}{\tau_{n}}}\right) \end{array}$$

Here we have an integration constant *A* with *n*_∞_ and τ_*n*_ both fixed parameters for the given fixed value of voltage *V*. If *n*(0) = 0, a reasonable assumption, given that in the voltage clamp protocols looked at, the initial holding voltage prior to the step is near −90 mV implying the channels are essentially closed, this simplifies to:


(13) $$\begin{array}{l} n\left(t\right)=n_{\infty }\left(1-e^{\frac{-t}{\tau_{n}}}\right). \end{array}$$

Equation (13) gives the time dependent open probability value for a single gate at a fixed voltage. Combining the results of Equations (10) and (13) yields the following complete description for *P*_*o*_(*t*):


(14) $$\begin{array}{l} P_{o}\left(t\right)={\left(n_{\infty }\left(1-e^{\frac{-t}{\tau_{n}}}\right)\right)}^{p}. \end{array}$$

**Figure 1:**
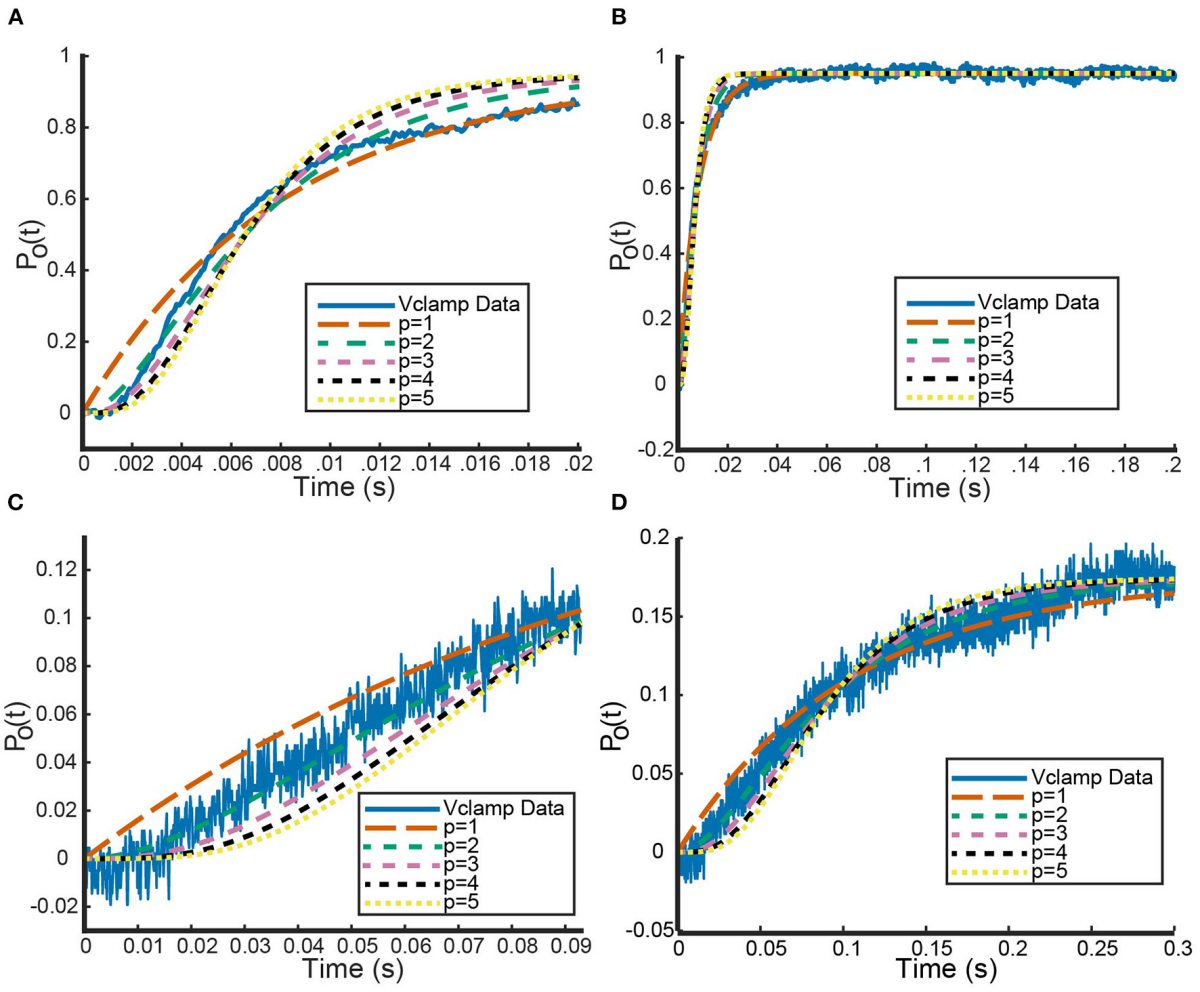
Sample fits of five curves of the form seen in Equation (14) to voltage clamp data (shown in blue) from Channelpedia (Ranjan et al., 2011, 2019). Each curve corresponds to a different value of *p*, the number of gates being used to fit the data. **(A,C)** A zoomed in version to make clear the performance of each model on capturing the sigmoid shape of the beginning portion of the data. **(B,D)** The whole timescale with each of the five fits. Curve colors are as follows: *p* = 1 (orange), *p* = 2 (green), *p* = 3 (pink), *p* = 4 (black), *p* = 5 (yellow). Curve dash sizing and spacing is given in figure legend. **(A,B)** correspond to a voltage step from −90 up to 20 mV while panels **(C,D)** correspond to a voltage step from −90 up to −30 mV.

Finally, using MATLAB's built in nonlinear least squares fitting method, we fit Equation 14, with different chosen values of *p*, to the transformed voltage clamp data (MATLAB, 2018). By setting bounds on the possible parameter values for *n*_∞_ (between 0 and 1) and τ_*n*_ (>0), the MATLAB function performs a parameter search that solves: $\text{min}\left[\Sigma {\left(P_{o}\left(t\right)-\text{data}\left(t\right)\right)}^{2}\right]$. In Figure 1, we see an example of how these curves fit a *K*_*V*_1.1 voltage clamp experiment with *V* = 20 mV (channels close to fully open) and *V* = −30*mV* (channels beginning to open) for different values of *p* from 1 to 5.

At initial glance, Figures 1B,D indicate that all powers of *p* generate roughly identical fits to the data with *p* = 1 performing marginally better. We compared the fits of the different power *p* curves across the entire data set using mean squared error (MSE) as our metric. Using voltage clamp recordings for six different cells, looking at data for six different value of *V*, we averaged the MSEs for all powers of *p*. This revealed that *p* = 1 generated the smallest MSE of 0.0006 and *p* = 5 the largest value of 0.0021 (Table 1).

**Table 1:** Different error metrics comparing homomeric *K*_*v*_ channel models with different gate numbers.

**p**	**Full**	**Short**	**Weighted**
1	0.0006	0.0028	0.28
2	0.0009	0.0006	0.06
3	0.0015	0.0004	0.04
4	0.0018	0.0004	0.04
5	0.0021	0.0004	0.04

The first column *p* is the number of gates and thus power used to fit the data. The Full column gives the MSE between each fit model and the entire dataset. The Short column gives the MSE between model and the data over the time interval where the channel has not yet opened. The Weighted column gives a weighted MSE over the whole time interval where the time component corresponding to when the channel is closed is weighted 100 times heavier.

However, zooming in on the data and the fits across a smaller range of time values shows an important detail to make note of Figures 1A,B clearly show clearly shows the shape of the data cannot be fit best with the simple exponential function that would result from setting *p* = 1. For each of the six cells, the voltage clamp data across all values of *V*, is seen to take on a sigmoid shape. This data, and the representative fits, suggests that modeling activation only *K*_*V*_ channels with the Hodgkin Huxley gating kinetics seen in Equation (11) will require more than 1 gate to correctly model all aspects of the known data.

To quantify this hypothesis, we looked at two other error metrics: a MSE over a shorter time interval and a weighted error. Looking at the MSE over the shorter time interval, defined to be the component of the data seen before channel opening, every power *p*>1 performed an order of magnitude (differing by a factor of 10) better than when *p* = 1 (Table 1). Although this analysis cannot distinguish between models with two or more gates, it does confirm that one gate is not sufficient for the shape of the data. This observation is recapitulated by the weighted error we computed. The weighted error is computed over the entire time interval by weighting the MSE over the short time interval 100 times more than the remainder of the time. The idea behind this weighted error is to see how well the model adheres to the sigmoid shape while still accounting for error over the remaining time. As with the short time interval error, *p* = 1 was an order of magnitude worse than every other power.

## 3. Results

### 3.1. *K*_*v*_1.1 and *K*_*v*_1.2 heteromers

Here we show the model's ability to reproduce certain 2013 results by the Al-Sabi group (Al-Sabi et al., 2013). In their work, they were able to concatenate subunits of *K*_*v*_1.1 and *K*_*v*_1.2 channels together into functional homomers and heteromers of every possible ratio. Then by way of voltage clamp experiments, they found each heteromer's and homomer's steady state open probability as a function of voltage. Their data points were then fit with the following open probability equation:


(15) $$\begin{array}{l} P_{open}\left(V\right)=\frac{1}{1+e^{\left(-\left(V-V_{.5}\right)/k\right)}} \end{array}$$

with different parameter values *V*_.5_ and *k* for each heteromer and homomer. Note that while Equation (15) is a function for the steady state probability of a given channel, it was fit assuming only one activating gate. Using the *V*_.5_ and *k* values for the *K*_*v*_1.1 and *K*_*v*_1.2 homomers from Al-Sabi et al. (2013), we calibrated the activating kinetics for the *K*_*v*_1.1 and *K*_*v*_1.2 gates to fulfill our four gate assumption. This was done by taking Equation (15) with parameters from Al-Sabi et al. (2013), and using the MATLAB nonlinear least squares fitting function to find the best fitting function of the form $P_{open}{\left(V\right)}^{4}$. The data points used in minimizing the least squares error were a discretized range of voltage values from −100 to 100 of step size 0.01. We arrived at the following steady state probabilities for *K*_*v*_1.1 and *K*_*v*_1.2 gates:


(16) $$\begin{array}{l} n_{1.1}\left(V\right)=\frac{1}{1+e^{\left(-\left(V+V_{1.1}\right)/k_{1.1}\right)}} \end{array}$$

(17) $$\begin{array}{l} n_{1.2}\left(V\right)=\frac{1}{1+e^{\left(-\left(V+V_{1.2}\right)/k_{1.2}\right)}} \end{array}$$

where *V*_1.1_ = 59.18, *k*_1.1_ = 15.61, *V*_1.2_ = 44.08, and *k*_1.2_ = 24.75. The parameter values for Equations (16) and (17) were found by using the described fitting method and the steady state probability curves for the *K*_*v*_1.1 and *K*_*v*_1.2 homomers from Al-Sabi et al. (2013). Note that variations in the values for the parameters *V*_1.1_, *k*_1.1_, *V*_1.2_, and *k*_1.2_ could possibly yield better fits to the Al-Sabi data as we were only able to fit to the available functional forms of their steady state curves. For clarity, this original set of parameters will be referred hereafter as the optimal, or best fitting, parameter set.

**Figure 2:**
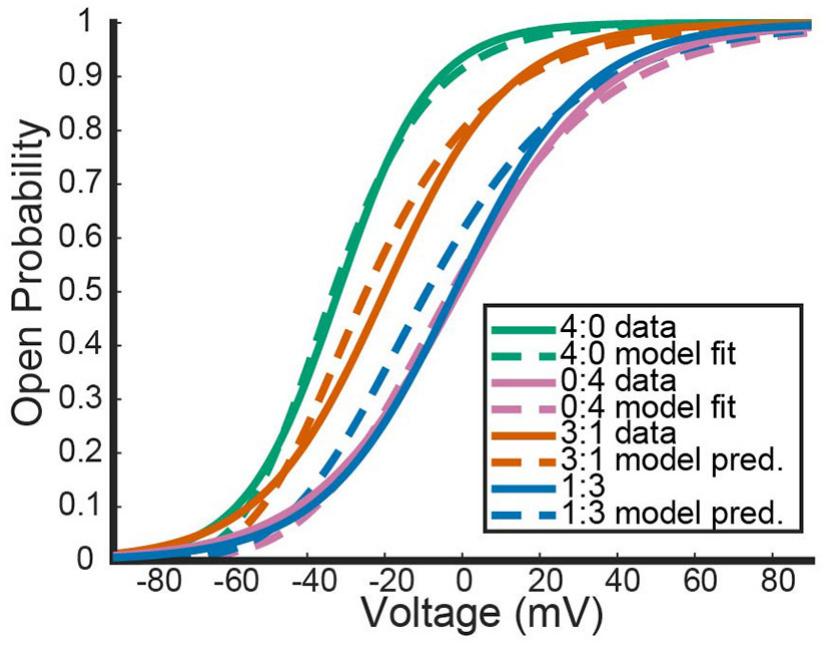
Comparisons between our mathematical framework predictions for *K*_*V*_1.1/1.2 heteromers and data from Al-Sabi et al. (2013). The solid curves are the plotted fits from Al-Sabi and the dashed curves are the results of the mathematical model. The green and pink curves correspond to the two different homomers with the dashed curve model outputs having been fit directly to Al-Sabi parameters as described above. The mean squared error (MSE) between 4:0 curves is 0.00022 and between 0:4 curves is 0.00033. The orange and blue curves are model prediction outputs for 3:1 and 1:3 *K*_*v*_1.1/1.2 heteromers using Equations (16) and (17) for the gating kinetics. The MSE for the 3:1 curve is 0.00091 and the MSE for 1:3 curves is 0.0018.

Recall that under the framework outlined above, a 3:1 *K*_*V*_1.1/1.2 heteromer will have steady state open probability $n_{1.1}^{3}n_{1.2}$ and a 1:3 heteromer will have open probability $n_{1.1}n_{1.2}^{3}$. While the 2:2 heteromer's probability curve could be generated in a similar manner, the data recorded in Al-Sabi et al. (2013) for the 2:2 heteromer was from a separate experiment and is therefore left out. Utilizing our fit curves, we compared the model predictions for the 3:1 and 1:3 heteromer's open probability against the fit summary curves from the Al-Sabi study. This is shown in Figure 2.

Figure 2 shows the model's attempt at replicating *K*_*V*_1.1/1.2 heteromeric steady state open probabilities. Most notably, the model does well at capturing the 3:1 heteromer steady state probability. This is highlighted by the mean squared error (MSE) for the 3:1 heteromer having the same order of magnitude as the 4:0 and 0:4 homomers. Additionally, we can see the model predicts some amount of nonlinearity in the shifts of probability curves moving from left to right. In Al-Sabi et al. (2013), they observed a much larger jump between the 4:0 and 3:1 curves compared with the difference in 1:3 and 0:4 curves. To explore this observation more concretely, we asked at what voltage do each of the different channel types (4:0, 3:1, 1:3, 0:4) achieve certain probabilities of being open. This was done by setting $n_{1.1}^{a}n_{1.2}^{4-a}$ equal to one of 10, 25, 35, 50, 75, or 80% and solving for *V* at all values of *a* between 0 and 4. This can be thought of as drawing horizontal lines through Figure 2 at a given probability and then plotting the intersection points with the steady state probability curves as a continuous function.

**Figure 3:**
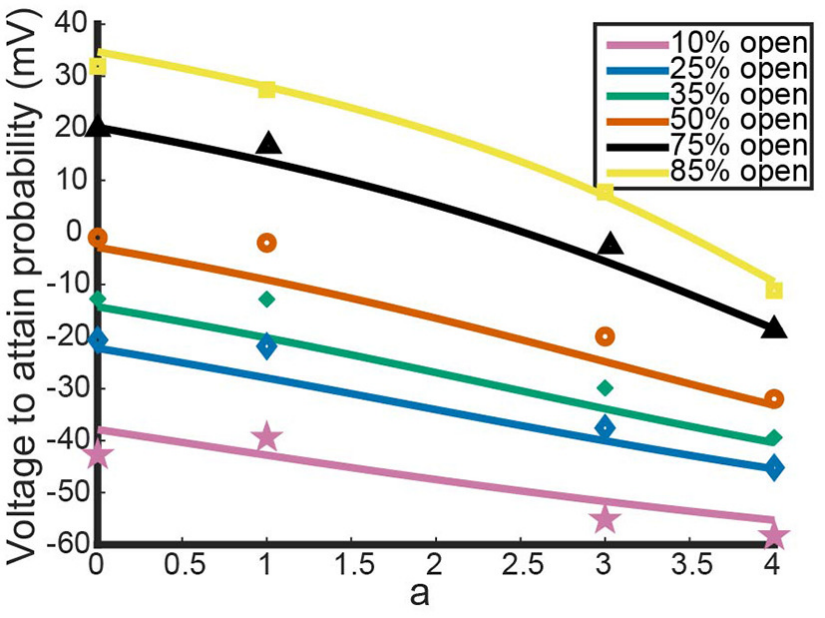
Model outputs showing the voltage at which a particular probability of opening is achieved. The x-axis denotes the proportion of subunits/gates that are *K*_*V*_1.1 vs. *K*_*V*_1.2 with *a* = 4 corresponding to a *K*_*V*_1.1 homomer with four subunits/gates. Each curve color corresponds to a different probability of opening from the model output. The data points are the Al-Sabi data points of each channel type for any given probability of opening and are colored accordingly. Although the non-integer values of *a* are irrelevant under the model framework, the curves are left for clarity of the trends moving from *a* = 4 to *a* = 0.

Figure 3 shows the voltage at which a specific probability of being open is reached as a function of *a*, the number of *K*_*v*_1.1 subunits. For instance, the red curve corresponds directly to the *V*_.5_ or 50% open probability of each channel type, with *a* = 4 giving the *V*_.5_ of *K*_*v*_1.1 homomers and *a* = 0 the *V*_.5_ of *K*_*v*_1.2 homomers. In Figure 3, we can clearly see the Al-Sabi data points showing the behavior that we hope the model replicates. The data points for each probability of opening show a much steeper increase going from four *K*_*v*_1.1 subunits to 3 in comparison with the minimal change going from 1 *K*_*v*_1.1 subunit to none. We see with this parameter set for *n*_1.1_(*V*) and *n*_1.2_(*V*) the model has this sharper change moving from *a* = 4 to *a* = 3 than moving from *a* = 1 to *a* = 0. However, this behavior is only present for the 50% and higher probability of opening curves, with the curves becoming more linear as the probability of opening is lowered.

**Figure 4:**
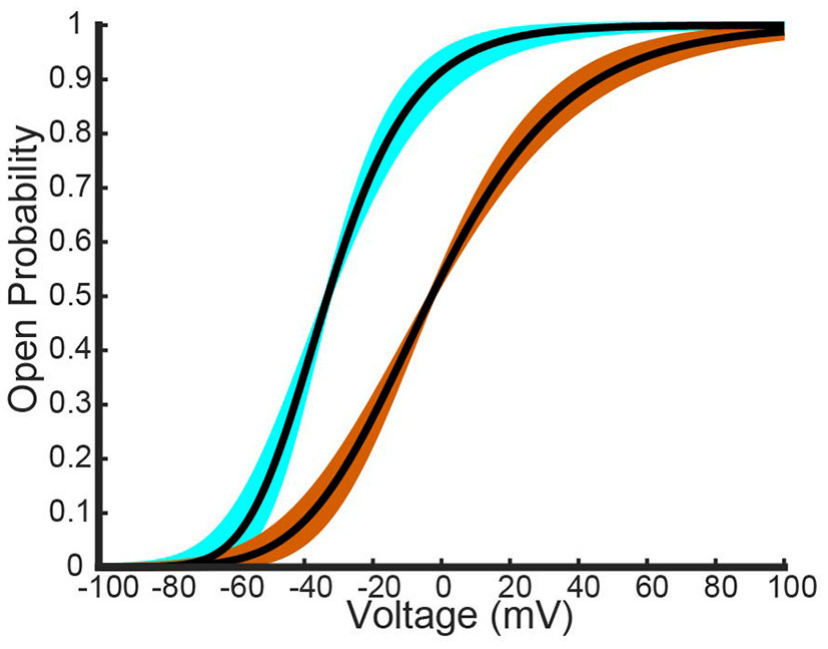
Different fits of a four gate model for *K*_*v*_1.1 and *K*_*v*_1.2 homomers to the Al-Sabi one gate model data. The best fit model generated from a nonlinear least squares fitting method is shown as a black curve for both channel types. The light blue curves are the other seven overlapping fit *K*_*v*_1.1 models that have the same order of magnitude as the best fit *K*_*v*_1.1 curve. The orange curves are the other overlapping eight fit *K*_*v*_1.2 models that have the same order of magnitude as the best fit *K*_*v*_1.2 curve.

Having noted above that this parameter set may not actually fit the data best, we perturbed *V*_1.1_, *k*_1.1_, *V*_1.2_, and *k*_1.2_ and examined the resulting model output. To perturb the parameters, we start with the original fit parameter set from above, increase or decrease *k*_1.*x*_, and then refit *n*_1.*x*_(*V*) to get a new *V*_1.*x*_, for *x* = 1, 2. Each *k*_1.*x*_ was both increased and decreased until the newly fit homomeric open probability curves produced a mean squared error with the Al-Sabi curves that was of a higher order magnitude (differencing by a factor of ten) than our original best fit model curves. This procedure yielded 8 different *K*_*v*_1.1 curves and 9 *K*_*v*_1.2 curves that had the same MSE as the optimal fit curves. These curves are shown below in Figure 4.

These different parameter fits yield 72 different model combinations to test. Here we present the model results using the two most insightful combinations: when the slope factors *k*_1.*x*_ are the closest and furthest apart.

**Figure 5:**
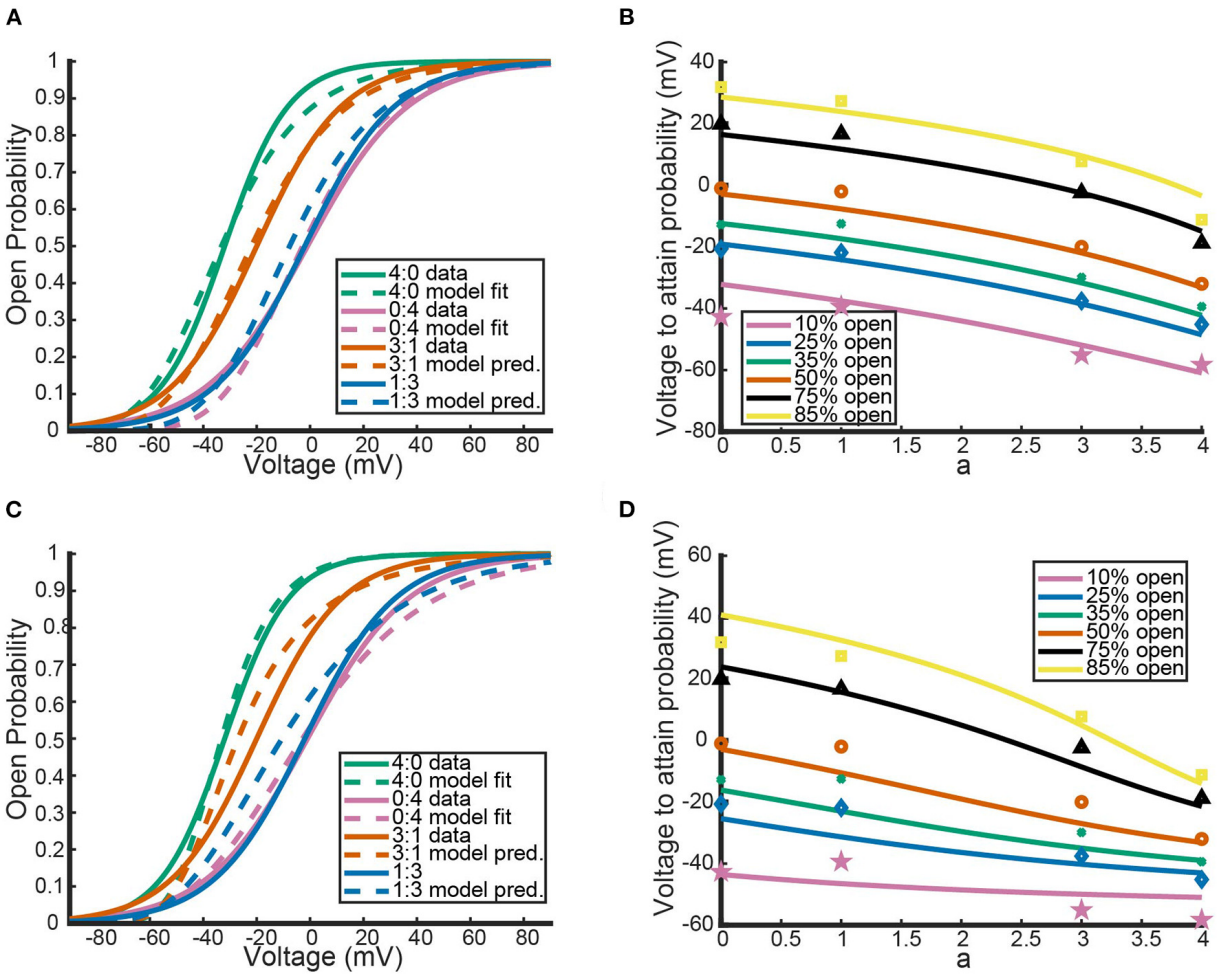
The model's performance relative to the Al-Sabi data using the *K*_*v*_1.1 and *K*_*v*_1.2 fit models with slope factors that are closest **(A,B)** and furthest **(C,D)** apart. Curve color and descriptions for **(A,C)** are identical to those given in Figure 2. Curve color and descriptions for **(B,D)** are identical to those given in Figure 3.

Figures 5A,B show the model results using homomeric open probability curves with the closest possible *k*_1.*x*_ values. This combination of parameter values resulted in heteromeric steady state probability curves that had more similar MSEs with the Al-Sabi data than those from the optimal parameter set. Additionally, Figure 5B shows a much better adherence to the observation that there are larger changes going from a 4:0 channel to a 3:1 channel than going from a 1:3 channel to a 0:4 channel. Whereas, the optimal parameter set generated curves that had the proper behavior only at higher probabilities of being open, the closest possible *k*_1.*x*_ parameter set had the correct curve concavity at all probabilities of being open.

In contrast, the outputs of the furthest apart possible *k*_1.*x*_ parameter set, shown in Figures 5C,D, do worse than the optimal parameter set. Not only are the 3:1 and 1:3 steady state open probability curves farther from the data than those of the optimal parameter set, the spacing of the steady state probability curves as seen in Figure 5D also performs worse than the optimal parameter set. At the highest probability of being open there is still a larger change from a 4:0 to a 3:1 channel than from a 1:3 to a 4:0 channel. This feature is reversed at lower probabilities of being open, which conflicts with the known shape of the data points. The two different parameter sets looked at here, found *via* our parameter search methodology, yield the possible extreme model outcomes in comparison to the data. We do not rule out the possibility of better or worse performing parameter sets, but with only the summary data curves available this analysis must suffice.

### 3.2. Model predictions: Three examples

With our heteromeric model, we can begin to answer questions surrounding experiments involving heteromeric activation-only K-channels. One such question regarding these channel types involves understanding the differences between cells expressing heteromeric channels and cells expressing only the homomeric counterparts. Are these two cell types noticeably different? More specifically, if a cell expresses DNA for two different K-Channel proteins, is it possible to discern in which ratios the subunits form channels? Examples of this experimental setup includes work with *K*_*v*_1.1, *K*_*v*_1.2, and *K*_*v*_1.1 mutant subunits, work with *K*_*v*_7.4 and *K*_*v*_7.5 subunits and work in *K*_*v*_7.2, *K*_*v*_7.3, and *K*_*v*_7.2 mutant systems (D'Adamo et al., 1999; Imbrici et al., 2008; Miceli et al., 2013, 2015, 2022; Mani et al., 2016; Hasan et al., 2017). In each system, the different subunit types were expressed either alone or together in a cell, and the resulting steady state open probability curves were found. It was shown that the coexpressed subunit cell's probability curves always lie between those of the homomeric probability curves. However, for each scenario, it was not clear if the coexpressed subunits are coming together randomly, assembling in a preferred heteromeric ratio, or assembling together preferring the homomeric configurations.

For each experiment, we computed the steady state open probability that satisfies the power 4 model assumption for each homomeric channel type. This was done using the experimentally derived *V*_.5_ and *k* values in the manner as described above in Section 3.1. With the fit equations for the different gate types, we could plot the steady state probability curves for the homomeric expression data, the coexpression data, and what the model predicts would be generated for subunits assembling only in certain heteromeric ratios or completely randomly. Probability curves for channels expressing only a specific heteromeric ratio are calculated directly according to the mathematical framework outlined above. To generate the predicted steady state probability curve stemming from a random assembly of the two subunits, we make two assumptions. First, we assume that the transcription and translation of the expressed DNAs occur with the same effectiveness for all DNA types. Since experimentally the two distinct DNA types are expressed at 50:50 ratios, this assumption implies there is an equal amount of each protein subunit type. The second assumption is that we define random assembly as a binomial process for choosing four subunits with 50% probability of choosing either type. Combining the model's steady state probability curves for all the heteromeric and homomeric channel types with this binomial distribution results in the following prediction for a steady state probability curve generated from random subunit assembly:


(18) $$\begin{array}{l} P_{rand}\left(V\right)=\frac{1}{16}P_{4:0}\left(V\right)+\frac{1}{4}P_{3:1}\left(V\right)+\frac{3}{8}P_{2:2}\left(V\right)+\frac{1}{4}P_{1:3}\left(V\right)\\ \;+\frac{1}{16}P_{0:4}\left(V\right). \end{array}$$

Here *P*_*x*:*y*_ denotes the probability curve for a channel with *x* subunits of the first type and *y* subunits of the second type. Note that a cell expressing only 2:2 heteromers will typically have an open probability curve that significantly overlaps with the random assembly curve, simply as a result of coefficients seen in Equation 18. The next subsections are the results of this analysis applied to multiple different experiments on *K*_*v*_1.1, *K*_*v*_1.2 heteromers, a *K*_*v*_7.4 and *K*_*v*_7.5 heteromeric experiment and multiple *K*_*v*_7.2 and *K*_*v*_7.3 heteromeric experiments. These results include looking at expression systems involving the wildtype subunits and different mutant types responsible for a multitude of neurological disorders.

#### 3.2.1. *K*_*v*_1.1, *K*_*v*_1.2, and *K*_*v*_1.1 mutant experiments

The first two experiments we looked at contain data for coexpression experiments with *K*_*v*_1.1 and *K*_*v*_1.2 wildtype DNA (D'Adamo et al., 1999; Imbrici et al., 2008).

**Figure 6:**
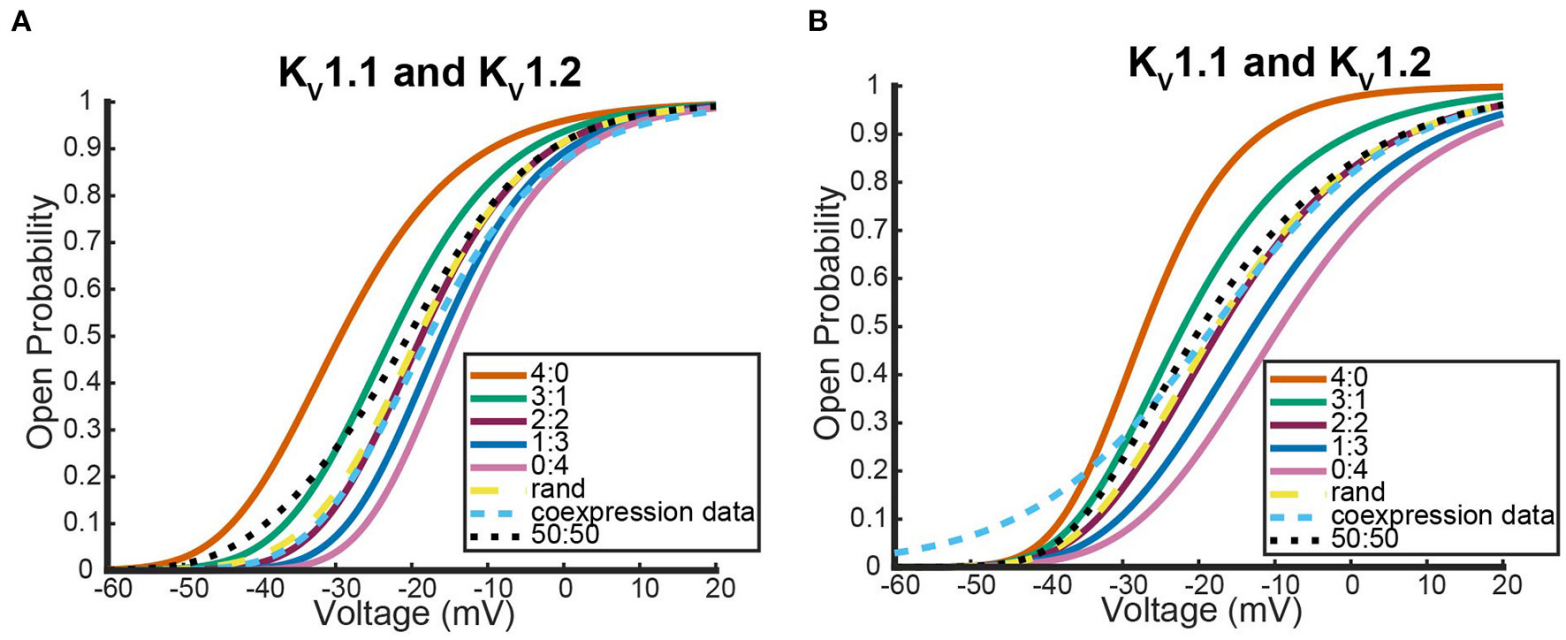
Steady state probability curves as a function of voltage for cells containing various channel types in two different experiments. The coexpression data and homomeric data for model fitting was taken from D'Adamo et al. (1999) **(A)** and Imbrici et al. (2008) **(B)**. The steady state probability curves presented here are for homomeric *K*_*v*_1.1 (4:0) and *K*_*v*_1.2 (0:4) channels, heteromeric *K*_*v*_1.1/1.2 channels (3:1, 2:2, 1:3), the model output of a random assembly of *K*_*v*_1.1 and *K*_*v*_1.2 subunits expressed in 50:50 ratio (rand), the data output of a 50:50 expression of *K*_*v*_1.1 and *K*_*v*_1.2 cDNAs (coexpression data), and a model output of an equal split between only homomeric channels (50:50). Curve color and type are shown in the legends. Homomeric channels and their related heteromers being looked at are also detailed in figure titles.

Figure 6 shows where the coexpression probability curves lie in relation to model predictions for two different *K*_*v*_1.1 and *K*_*v*_1.2 experiments. In both experiments, we see the coexpression curve overlapping with the 2:2 heteromeric model prediction and the random assembly model prediction curves for a wide range of voltages. Although more data would be necessary to distinguish which curve the coexpression data most resembled, we show the model predicts that *K*_*v*_1.1 and *K*_*v*_1.2 subunits prefer to assemble in a 2:2 conformation or completely randomly. This result is in stark contrast to what happens when *K*_*v*_1.1 subunits are coexpressed with a variety of different *K*_*v*_1.1 mutant types. We looked at the following three neurological disease associated *K*_*v*_1.1 mutants: F303V, A261T, and P405S (Hasan et al., 2017; Miceli et al., 2022).

**Figure 7:**
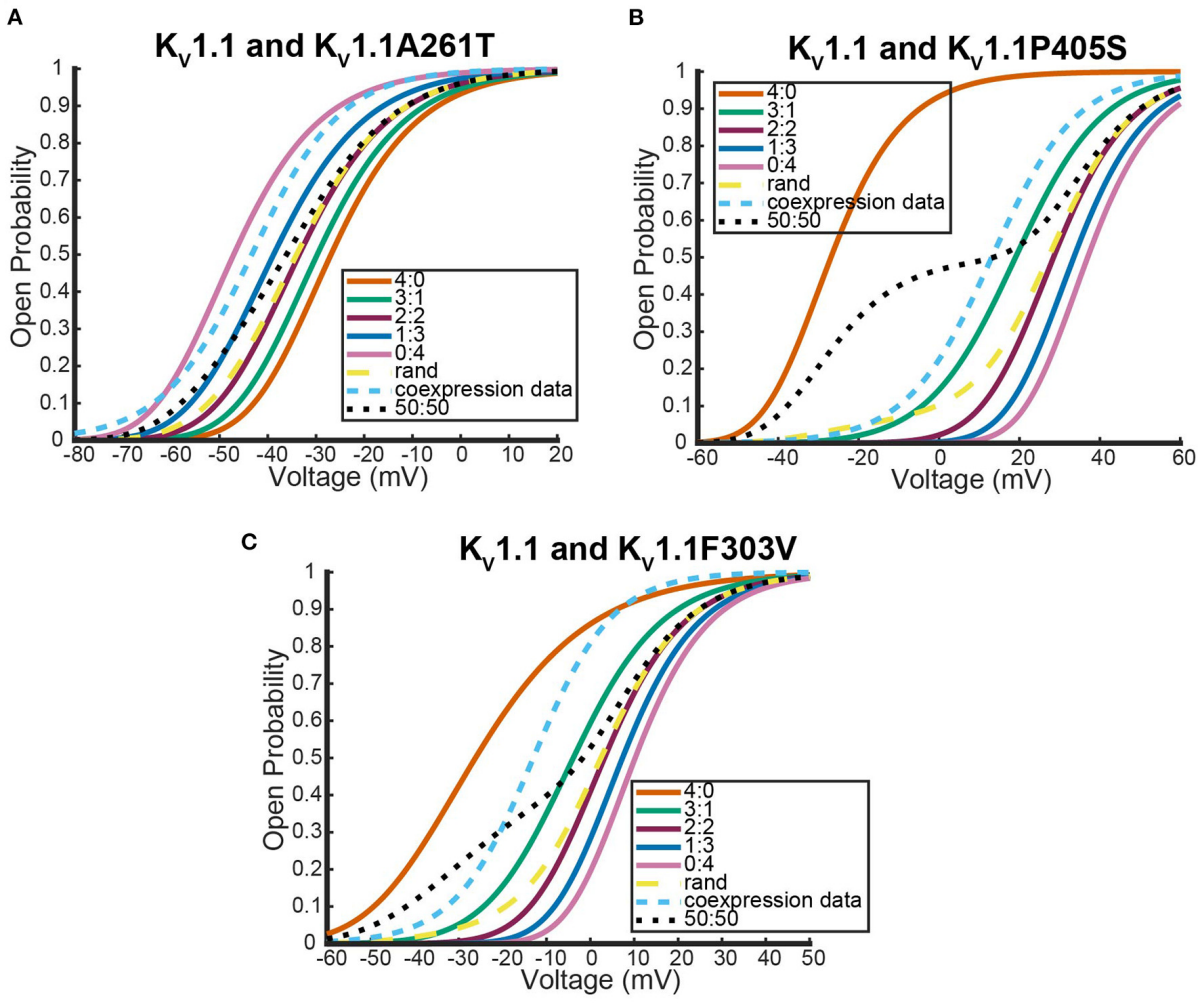
Steady state probability curves as a function of voltage for cells containing various channel types in three different experiments. The coexpression data and homomeric data for model fitting was taken from Miceli et al. (2022) **(A,B)** and Hasan et al. (2017) **(C)**. The steady state probability curves presented here are for homomeric *K*_*v*_1.1 (4:0) and *K*_*v*_1.1 mutant (0:4) channels, heteromeric assembly of *K*_*v*_1.1 and *K*_*v*_1.1 mutant subunits (3:1, 2:2, 1:3), the model output of a random assembly of *K*_*v*_1.1 and *K*_*v*_1.1 mutant subunits expressed in 50:50 ratio (rand), the data output of a 50:50 expression of *K*_*v*_1.1 and mutant *K*_*v*_1.1 subunits (coexpression data), and a model output of an equal split between only homomeric channels (50:50). Curve color and type are shown in the legends. Homomeric channels and their related heteromers being looked at are also detailed in figure titles.

Unlike the *K*_*v*_1.1 and *K*_*v*_1.2 wildtype coexpression experiments shown in Figure 6, we see in Figure 7 that each coexpression data curve does not overlap with the random assembly curves. In fact, for each mutant, the coexpression probability curves appear to have a preference for a conformation containing three subunits of one type and one of the other. It is important to observe that this preferred ratio is not always with three wildtype subunits as might be initially assumed. In the case of the *K*_*v*_1.1 and *K*_*v*_1.1*A*261*T* mutant coexpression data, the curve lies between the 0:4 mutant homomeric curve and the 1:3 heteromeric curve (Figure 7A). Meanwhile, the other mutant experiments both had coexpression data curves that were between the 4:0 wildtype homomeric and the 3:1 heteromeric curves (Figures 7C,D). While it is unclear why certain mutations would cause preferences for a 3:1 conformation and other mutations would prefer a 1:3 heteromic configuration; we highlight that in all cases the coexpression data lies between the heteromer and homomer curves that activate at the earliest voltages.

#### 3.2.2. *K*_*v*_7.4 and *K*_*v*_7.5 experiments

**Figure 8:**
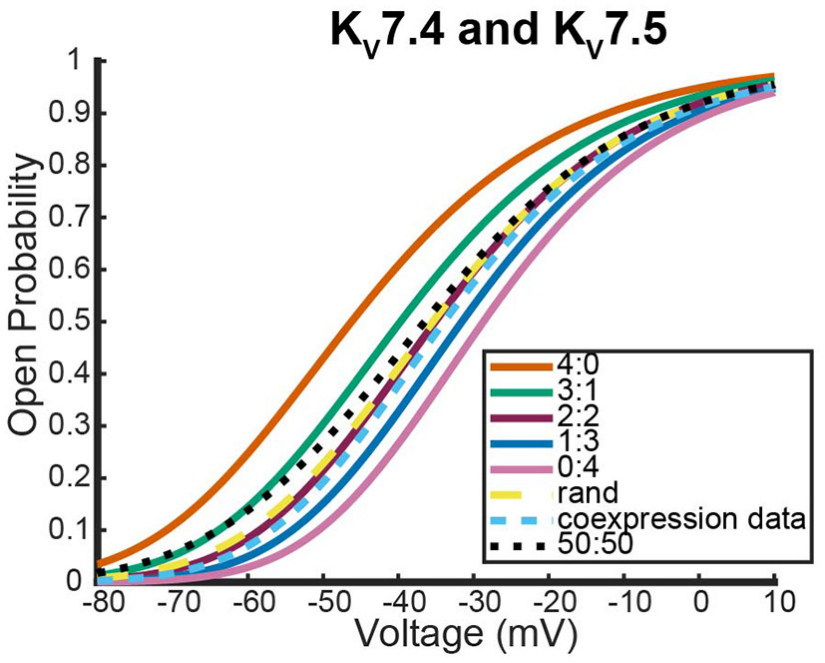
Steady state probability curves as a function of voltage for cells containing various channel types. The coexpression data and homomeric data for model fitting was taken from Mani et al. (2016). The steady state probability curves presented here are for homomeric *K*_*v*_7.4 (4:0) and *K*_*v*_7.5 (0:4) channels, heteromeric *K*_*v*_7.4/7.5 channels (3:1, 2:2, 1:3), the model output of a random assembly of *K*_*v*_7.4 and *K*_*v*_7.5 subunits expressed in 50:50 ratio (rand), the data output of a 50:50 expression of *K*_*v*_7.4 and *K*_*v*_7.5 subunits (coexpression data), and a model output of an equal split between only homomeric channels (50:50). Curve color and type are shown in the legend. Homomeric channels and their related heteromers being looked at are also detailed in figure title.

Here we address the application of our model to a coexpression experiment by Mani et al. (2016) involving *K*_*v*_7.4 and *K*_*v*_7.5 DNA. Currently, there is no experiment similar to the work by Al-Sabi et al. (2013) for us to validate the heteromeric framework against for *K*_*v*_7.4 and *K*_*v*_7.5 heteromeric channels. However, both *K*_*v*_7.4 and *K*_*v*_7.5 form homomeric tetramers lacking noticeable inactivation thereby satisfying the necessary assumptions to be modeled identically to the *K*_*v*_1.1 and *K*_*v*_1.2 system (Lipinsky et al., 2020; Harding et al., 2022). Under the assumption that this framework is a reasonable approximation for *K*_*v*_7.4 and *K*_*v*_7.5 heteromers, we look at a coexpression experiment of *K*_*v*_7.4 and *K*_*v*_7.5 DNA done by Mani et al. (2016) with the results shown in Figure 8.

As was seen with the *K*_*v*_1.1 and *K*_*v*_1.2 wildtype experiments, the coexpression data overlaps with the 2:2 heteromer and the random assembly curves (Figure 8). This result is especially striking since it has been shown experimentally that unique characteristics of M-currents can be replicated with 2:2 *K*_*v*_7.4 and *K*_*v*_7.5 heteromers (Brueggemann et al., 2020).

#### 3.2.3. *K*_*v*_7.2, *K*_*v*_7.3, and *K*_*v*_7.2 mutant experiments

The final system we looked at that had similar coexpression experiments with wildtype heteromers that are not known to possess meaningful inactivation kinetics involved *K*_*v*_7.2, *K*_*v*_7.3, and *K*_*v*_7.2 mutants (Lipinsky et al., 2020; Harding et al., 2022). The first data looked at were from a coexpression experiment between wildtype *K*_*v*_7.2 and *K*_*v*_7.3 to see how *K*_*v*_7.2 heteromerizes (Miceli et al., 2015).

**Figure 9:**
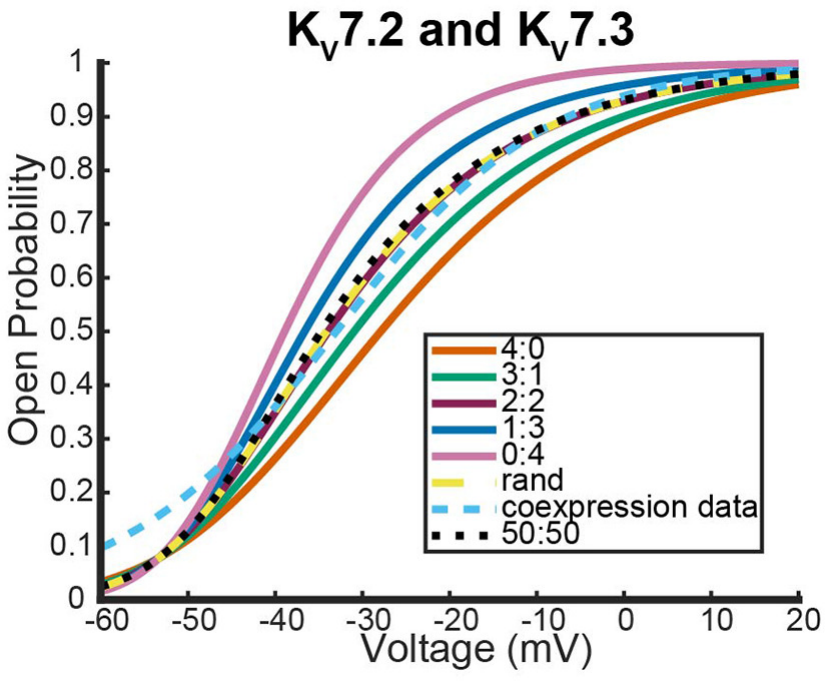
Steady state probability curves as a function of voltage for cells containing various channel types. The coexpression data and homomeric data for model fitting was taken from Miceli et al. (2015). The steady state probability curves presented here are for homomeric *K*_*v*_7.2 (4:0) and *K*_*v*_7.3 (0:4) channels, heteromeric *K*_*v*_7.2/7.3 channels (3:1, 2:2,1:3), the model output of a random assembly of *K*_*v*_7.2 and *K*_*v*_7.3 subunits expressed in 50:50 ratio (rand), the data output of a 50:50 expression of *K*_*v*_7.2 and *K*_*v*_7.3 subunits (coexpression data), and a model output of an equal split between only homomeric channels (50:50). Curve color and type are shown in the legend. Homomeric channels and their related heteromers being looked at are also detailed in figure title.

As was seen with the other two systems we looked at, expression of the two different wildtype DNAs results in a steady state probability curve that overlaps with the 2:2 and random assembly steady state probability curves for a large range of voltages (Figure 9). Once again, when we switched to coexpression experiments involving mutant subunit DNA the results were noticeably different. We looked at the coexpression of *K*_*v*_7.2 and four different *K*_*v*_7.2 mutants: R201H, R144Q, R213Q, and R213W (Miceli et al., 2013, 2015).

**Figure 10:**
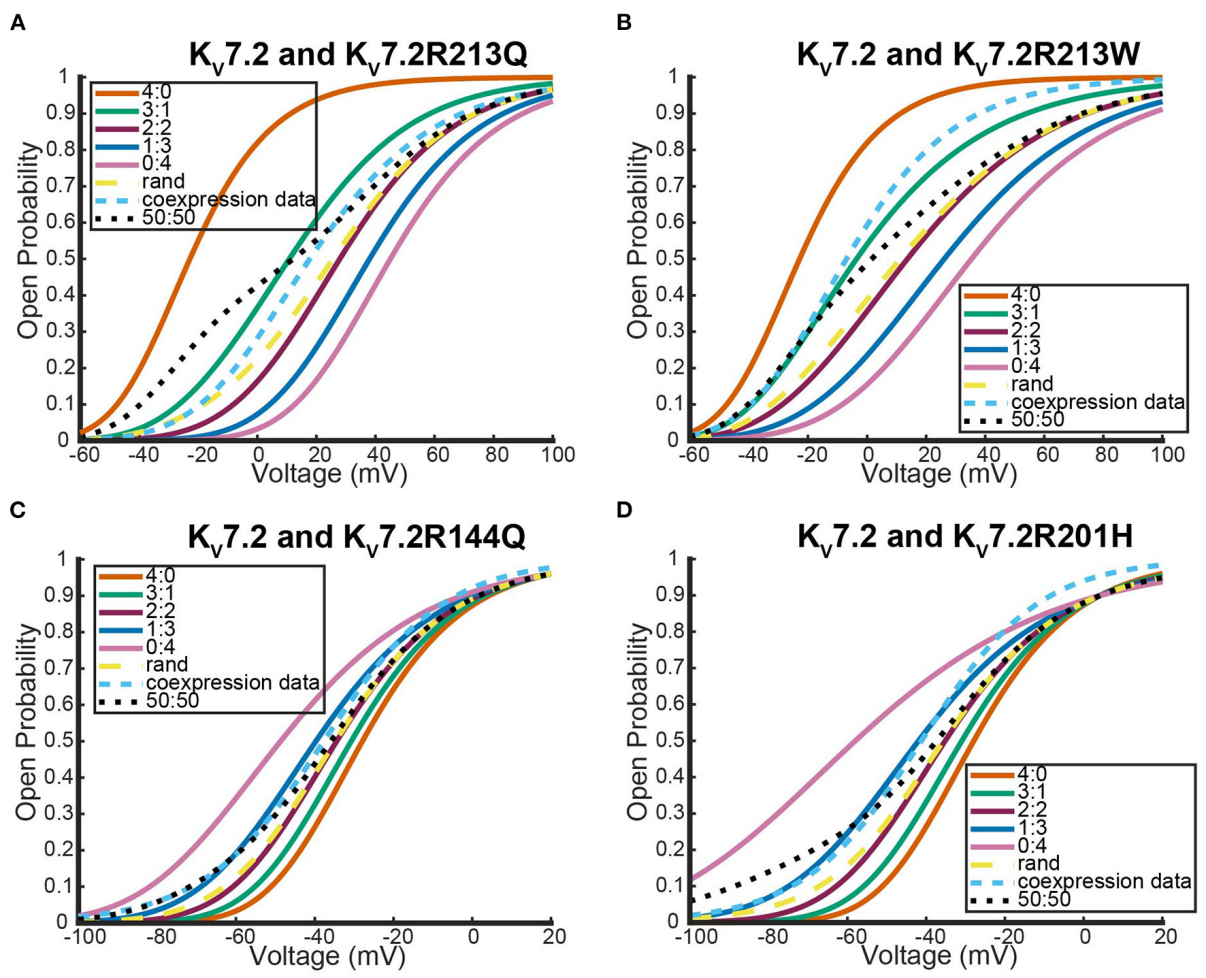
Steady state probability curves as a function of voltage for cells containing various channel types in four different experiments. The coexpression data and homomeric data for model fitting was taken from **(A,B)** Miceli et al. (2013) and **(C,D)** Miceli et al. (2015). The steady state probability curves presented here are for homomeric *K*_*v*_7.2 (4:0) and *K*_*v*_7.2 mutant (0:4) channels, heteromeric assembly of *K*_*v*_7.2 and *K*_*v*_7.2 mutant subunits (3:1, 2:2, 1:3), the model output of a random assembly of *K*_*v*_7.2 and *K*_*v*_7.2 mutant subunits expressed in 50:50 ratio (rand), the data output of a 50:50 expression of *K*_*v*_7.2 and mutant *K*_*v*_7.2 subunits (coexpression data), and a model output of an equal split between only homomeric channels (50:50). Curve color and type are shown in the legends. Homomeric channels and their related heteromers being looked at are also detailed in figure titles.

Interestingly, as was seen with *K*_*v*_1.1 and its mutants, each *K*_*v*_7.2 and *K*_*v*_7.2 mutant coexpression experiment generated a steady state probability curve near or overlapping a curve corresponding to a 3 to 1 ratio of subunits. As before, this stoichiometric ratio of subunits is not a result of preference for wildtype subunits, but a preference toward activating at earlier or at more negative voltages. This is observable in Figures 10A,B by seeing that when the mutant subunit has a *V*_.5_ to the right of the *V*_.5_ of *K*_*v*_7.2, the data lies near the 3:1 heteromer. On the other hand, if the mutant subunit has a more negative *V*_.5_ than the *K*_*v*_7.2 wildtype subunit, the data overlaps with the 1:3 heteromer (Figures 10C,D).

## 4. Discussion

Heteromeric *K*_*V*_ channels have garnered significant attention in recent years. Some heteromers have been shown to be the specific target of conotoxins while other heteromers are hypothesized to be the primary cause of certain currents (Cordeiro et al., 2019; Brueggemann et al., 2020). This uptick in discoveries has resulted in an abundance of new questions about heteromers' importance, how their kinetic properties relate to homomeric channels, the subunit ratios in which they form, and if they are present in natural systems. Despite mechanistic differential equation modeling being a common technique for answering tough experimental neuroscience questions, little modeling of heteromeric *K*_*V*_ channels has been done. Current modeling work for heteromeric experiments either looks at different channel families or lacks the necessary known biological specificity (Cheng et al., 2007; Miceli et al., 2013, 2015; Benndorf et al., 2022).

Here we presented a novel heteromeric *K*_*V*_ channel model framework that captures both α-subunit number and type. We began by outlining and defending key model assumptions. We then examined the framework's effectiveness and ability to reproduce key observations using a *K*_*V*_1.1/1.2 concatemer experiment. Finally, using results of various cDNA coexpression experiments, we used the model to make unique observations about the assembly of K-Channel α-subunits and their preferences toward certain stoichiometries.

### 4.1. Model and assumptions

We have proposed a new model framework for heteromeric K-channels with little to no inactivation kinetics. The model stipulates that each α-subunit present in the heteromer contributes one mathematical gate to the overall open probability. Equations for each gate type are derived from voltage clamp experiments performed on homomeric channels. With this model structure, once two homomers' steady state open probabilities are known, we can predict the kinetics for a heteromer with any ratio of these two homomers' subunits.

Key model assumptions were investigated and subsequently justified by comparing the framework to a Markov model and by analyzing time dependent voltage clamp data. To address our assumption that each subunit contributes one gate's worth of kinetics, we compared the Hodgkin-Huxley model with known ion channel Markov models. This analysis showed that for each ratio of subunits, our heteromeric model has a Markov model analog of four independently activating protein subunits that can switch between an open and closed state. Although the work here was done assuming each homomer is adequately modeled by four independent subunits that each have only two states, the only necessary condition for the model framework is subunit independence. For example, Markov models for homomeric potassium channels where each independent subunit has multiple closed states are given as possible models for *I*_*Ks*_, the slow delayed rectifier *K*^+^ current (Silva and Rudy, 2005; Keener, 2009). If a model of this type was required for one, or both, of the homomeric channels, it is possible to write a Markov model for 3:1, 2:2, and 1:3 heteromeric channels that still adhere to the one subunit to one “gate” hypothesis. In this case, the term “gate” is slightly misleading as the full Markov model no longer reduces to the Hodgkin and Huxley equations but something more complex. The derivation of the invariant manifold for the homomeric channel with subunits containing multiple closed states can be found in Keener (2009, 2010). Channels whose subunits undergo conformational changes in a cooperative manner break the independence assumption and therefore are not within the scope of the hypothesized model.

Continued exploration of the hypothesis that four gates are necessary and sufficient was done by using a publicly available database of voltage clamp experiments on *K*_*V*_1.1 channels (Ranjan et al., 2011, 2019). Looking at multiple different cell voltage clamp recordings, each at multiple voltage steps, we showed that more than one gate is required to replicate the sigmoid shape of the time dependent data (Ranjan et al., 2011, 2019). We were unable to show that four gates provided the best overall fit to the data, but we did show that a single Hodgkin-Huxley like gating equation results in an exponential shape that cannot account for the delay of activation seen in the data (Figure 1). This observation is in agreement with previous work on *K*_*V*_1.4 and *K*_*V*_11.1 (hERG) channels that detailed the importance of including more than one gate, and in some cases more than one activating gate type (Wang et al., 1997; Bett et al., 2011). This question regarding the cause of the sigmoidicity of the data remains open and warrants future examination; but for future researchers using Hodgkin-Huxley like gating equations to model channel kinetics, we stress that the full time dependent data from voltage clamp experiments should be used to fit kinetic properties as this provides critical insight into the number of activating steps occurring.

### 4.2. *K*_*V*_1.1/1.2 concatemeric experiment

To test the accuracy of our model, we used results from a study that looked at the open probability curves and TEA sensitivity of *K*_*V*_1.1/1.2 concatemers (Al-Sabi et al., 2013). Using gating equations generated from the study's homomeric *K*_*V*_1.1 and *K*_*V*_1.2 steady state probability curves, we generated probability curves for each heteromic channel type (3:1, 2:2, and 1:3). The model outputs aligned well with the data, although the model predicts activation at earlier voltages than the data suggests. This result hints at either external factors influencing channel opening or a dependence between subunits. To verify this hypothesis, future studies should perform similar concatemeric experiments with other activating only heteromers to see if this behavior is standard across K-channel families.

The second result from comparisons to this experiment was how the model captured shifts in steady state probability curves as more *K*_*V*_1.2 subunits were added. In the study it was observed there is a noticeably larger shift to activating later when transitioning from a $K_{1.1/1.2}^{4:0}$ homomer to a $K_{1.1/1.2}^{3:1}$ heteromer than when shifting from $K_{1.1/1.2}^{1:3}$ heteromer to a $K_{1.1/1.2}^{0:4}$ homomer (Al-Sabi et al., 2013). Our model shared this phenomenon, although it was more pronounced for the larger probabilities of opening.

A primary limitation for this particular analysis was data availability. Only summary parameters that generated the steady state data curves were available and not the actual data itself. To combat this issue, we generated a few different fits to the homomeric steady state curve data and explored the newly fit models' performances. Here we presented two model extremes: one where the *K*_*V*_1.1 and *K*_*V*_1.2 homomeric model steady state curves were as close in slope factor (*k*_1.1_, *k*_1.2_) as possible, and the other where the slope factors were as far apart as possible. Only at the extreme where the homomeric slopes were fit to be far apart did the model framework begin to lose accuracy in both the steady state probability curves it generated and the spacing between said curves. In fact, heading toward the other extreme, slopes closer together, there was improvement in both heteromeric curve accuracy and the curve spacing accuracy. Thus, for a wide range of fitted homomeric model curves, our framework was able to mimic the steady state probability curves and the key observations about the relationship between them from Al-Sabi et al. (2013).

The second issue arising from a lack of data availability involved investigation of the time component of activation for these heteromeric concatemeric channels. In the work by Al-Sabi et al. (2013), time constants at a few select voltage values for all stoichiometric ratios are given. While this information could be used to fit a function of τ_*n*_(*V*), the voltage dependent time constant, for the homomeric *K*_*v*_1.1 and *K*_*V*_1.2 models, there are issues drawing comparisons between the heteromeric time constants recorded in their work and model outputs. Under the proposed model, the heteromeric channels have two time components associated with their activation, one for each subunit type. This hypothesizes that the time course is the result of a double exponential and not the single exponential that was used to fit the heteromeric data. Therefore, there is no way to make direct comparisons between the model's prediction of the time constants of activation to the published time constant data. The limitations with the steady state analysis and with performing time constant analysis can both be resolved if the full time dependent voltage clamp data of the heteromeric channels is known. This then allows for direct comparisons between raw data and model outputs by simulating the heteromeric models through identical experimental protocols.

### 4.3. Coexpression experiments

We looked at three different systems of voltage gated potassium channels where DNA's for two different subunits, known to form heteromeric tetramers, were expressed in the same cell. By taking the steady state probability curves generated by the experiments and comparing them to our model's predictive outputs, we examined if certain subunits have preferred configurations and if so, what they are. With each of the different systems we studied, our model consistently predicted only two preferred configurations. The outcome was completely dependent on if the coexpression experiment was between two wildtype DNAs or if one DNA was for a mutant subunit responsible for certain neurological disorders.

If both cDNA's being expressed were wildtype, then the resulting coexpressed steady state probability curve overlapped the 2:2 heteromer and random assembly curves. As noted earlier, the inability to distinguish the coexpression curve from either of these curves is not surprising due to the coefficients in Equation (18). The model therefore predicts wildtype subunits assemble either with a preference toward the 2:2 configuration or, as has been suggested by some, random assembly with no stoichiometric preference (Miceli et al., 2013, 2015). More data and refined experimental techniques would be required to distinguish both mathematically and experimentally how precisely these wildtype subunits are assembling.

For each of the mutant coexpression experiments, the model indicates a clear preference away from the random assembly curve. In all cases the coexpression curve is found to be near a three to one subunit ratio curve where the three subunits correspond to whichever homomeric channel activates earlier. Critically, this predicts in some cases there is a preference toward channels assembling with more mutant subunits, while other mutations skew the assembly toward having one or fewer mutant subunits. This prediction could be the result of multiple different mechanisms. One possible mechanism is that upon 50–50 expression, the DNA is translated and transcribed equally, but not all subunit ratios form functional channels thereby weighting the steady state probability curve toward one side. A second option is the two DNA types are again translated and transcribed equally, but the proteins have a preferred ratio that they assemble in due to structure or compatibility. A third hypothesis for this outcome could be that the “translated and transcribed equally” assumption does not hold. Regardless of the mechanisms at play here, the model predicts that mutant and wildtype subunits for *K*_*v*_ channels lacking inactivation kinetics assemble in a non-Bernoulli manner.

## 5. Conclusion

We have constructed a new mechanistic model framework for heteromeric voltage gated potassium channels. Based on justification of assumptions, model validation against known experimental results, and exploration of the possible questions the model can address; we claim this model is an excellent starting point for heteromeric *K*_*V*_ channel models moving forward.

## Data availability statement

The datasets presented in this study can be found in online repositories. The names of the repository/repositories and accession number(s) can be found at: https://github.com/keesmcgahan/Heteromeric-K-Channels.

## Author contributions

KM: manuscript writing, model creation, model analysis, and coding. JK: manuscript editing, supervision, and guidance with model creation and analysis. All authors contributed to the article and approved the submitted version.

## Conflict of interest

The authors declare that the research was conducted in the absence of any commercial or financial relationships that could be construed as a potential conflict of interest.

## Publisher's note

All claims expressed in this article are solely those of the authors and do not necessarily represent those of their affiliated organizations, or those of the publisher, the editors and the reviewers. Any product that may be evaluated in this article, or claim that may be made by its manufacturer, is not guaranteed or endorsed by the publisher.
